# Wound healing responses of urinary extravasation after urethral injury

**DOI:** 10.1038/s41598-023-37610-2

**Published:** 2023-06-30

**Authors:** Taiju Hyuga, Kota Fujimoto, Daiki Hashimoto, Kazuya Tanabe, Taro Kubo, Shigeru Nakamura, Yuko Ueda, Eriko Fujita-Jimbo, Kazuhiro Muramatsu, Kentaro Suzuki, Hitoshi Osaka, Shinichi Asamura, Kimihiko Moriya, Hideo Nakai, Gen Yamada

**Affiliations:** 1 Department of Developmental Genetics Institute of Advanced Medicine, Wakayama Medical University, Kimiidera 811-1, Wakayama City, Wakayama 641-8509 Japan; 2 Department of Pediatric Urology, Jichi Medical University Children’s Medical Center Tochigi, Yakushiji 3311-1, Shimotsuke City, Tochigi 329-0498 Japan; 3 Department of Plastic and Reconstructive Surgery, Wakayama Medical University, Kimiidera 811-1, Wakayama City, Wakayama 641-8509 Japan; 4 Department of Urology, Wakayama Medical University, Kimiidera 811-1, Wakayama City, Wakayama 641-8509 Japan; 5 Department of Pediatrics, Jichi Medical University School of Medicine, Yakushiji 3311-1, Shimotsuke City, Tochigi 329-0498 Japan; 6 Faculty of Life and Environmental Sciences, University of Yamanashi, Takeda 4-4-37, Kofu City, Yamanashi 400-8510 Japan

**Keywords:** Urology, Paediatric urology, Urethra

## Abstract

The post-surgical fluid leakage from the tubular tissues is a critical symptom after gastrointestinal or urinary tract surgeries. Elucidating the mechanism for such abnormalities is vital in surgical and medical science. The exposure of the fluid such as peritonitis due to urinary or gastrointestinal perforation has been reported to induce severe inflammation to the surrounding tissue. However, there have been no reports for the tissue responses by fluid extravasation and assessment of post-surgical and injury complication processes is therefore vital. The current model mouse study aims to investigate the effect of the urinary extravasation of the urethral injuries. Analyses on the urinary extravasation affecting both urethral mesenchyme and epithelium and the resultant spongio-fibrosis/urethral stricture were performed. The urine was injected from the lumen of urethra exposing the surrounding mesenchyme after the injury. The wound healing responses with urinary extravasation were shown as severe edematous mesenchymal lesions with the narrow urethral lumen. The epithelial cell proliferation was significantly increased in the wide layers. The mesenchymal spongio-fibrosis was induced by urethral injury with subsequent extravasation. The current report thus offers a novel research tool for surgical sciences on the urinary tract.

## Introduction

The post-surgical fluid leakage after the gastrointestinal or urinary tract surgeries is critical symptom for the operated patients. The mechanism for the occurrence of such abnormalities remains unexplored^1^.

Urethral mechanical injuries were observed in blunt trauma and after surgical operations including the hypospadias repair^2–5^. Hypospadias accounts for a substantial proportion of pediatric urology cases. Its management is complex still lacking standardization as surgical techniques^6,7^. It has been widely known that these urethral mechanical injuries cause the urethral stricture disease, often leading to further urethral reconstruction surgeries^8^. The histological descriptions on the healing process after urethral injury have been reported as its mesenchymal spongio-fibrosis^1^. Furthermore, various clinical complications have been suggested for urinary extravasation^9^.

The healing processes after urethral injury frequently induce urethral stricture. The processes leading to fibrosis have been previously reported in other cases for extravasations^10^. It has been still unknown for the mechanism of such process after urethral injury and the subsequent spongio-fibrosis. In addition, the effects of the extravasation of fluids have not been virtually investigated and most reports on fluid extravasation only showed the subsequent systemic or local inflammations^8,11^. It has been only speculated, that spongio-fibrosis is associated with urethral stricture after the injury with urine leakage.

The purpose of this study is to investigate the effect of the urinary extravasation of the urethral injuries. The process of spongio-fibrosis was reproduced in detail for the first time by the current experimental system. The status of epithelial and mesenchymal cell proliferation after urethral injury was also evaluated. This is the first comprehensive analysis on the urinary extravasation which frequently leads to spongio-fibrosis and urethral stricture.

## Results

### The establishment of murine urethral injury (trauma) and extravasation model

**Figure 1:**
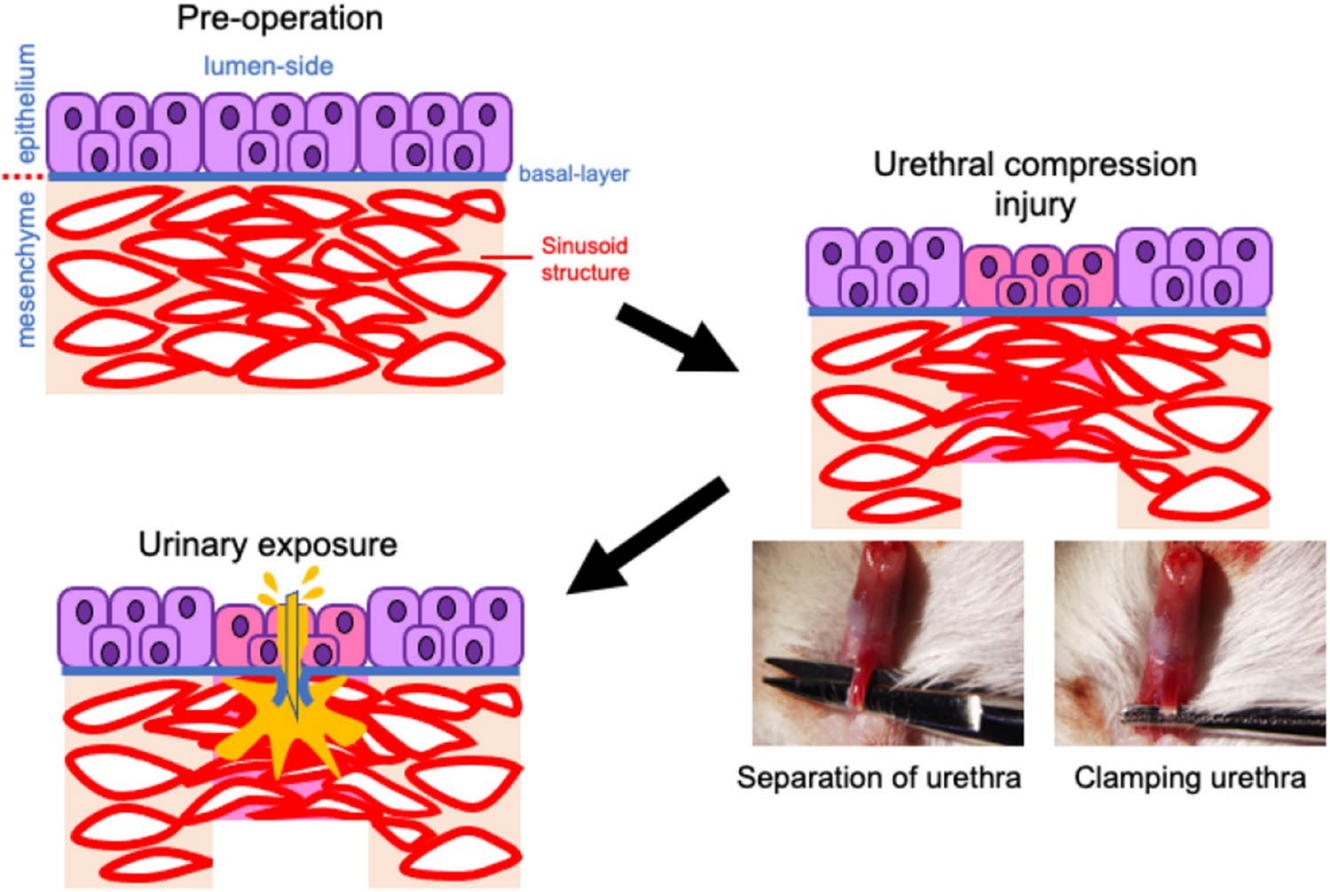
The schema of the murine urethral injury model is shown. The urethral epithelium is formed by pseudostratified columnar epithelium (shown by purple-colored epithelial cells). The urethra was separated from the corpus cavernosum, and the compression injury using mosquito forceps was suffered (right bottom rows). The isolated 0.2 ml urine was injected from the lumen of urethra using 29 gauge needle and the urethral mesenchyme was thus exposed to urine (urinary extravasation).

Murine urethral injury model was established. After general anesthesia, mice were placed in supine position. The urethra was separated from the corpus cavernosum using micro scissors, and was clamped with mosquito forceps (Fig. 1; the lower right photos).

Next, urinary extravasation procedure was developed. After clumping, lower abdominal small incision was placed and bladder was exposed. Urine was isolated with bladder puncture by 29 gauge needle and the urethra was punctured from luminal side. The isolated 0.2 ml urine was injected from the lumen of urethra utilizing the same needle and the urethral mesenchyme was thus exposed to urine. Since the clamped site and the needle position was also visible due to the thin urethra, injection into the clamped site was confirmed. The urethral epithelium is formed by pseudostratified columnar epithelium (Fig. 1; shown by purple-colored epithelial cells).

### The urethral mesenchymal lesions by the injury with urinary extravasation

In the current study, three experimental Groups were categorized.

Group1 corresponds to urethral injury with urinary extravasation.

Group2 corresponds to urethral injury without urinary extravasation.

Group3 corresponds to sham operation model in which urethra was separated from corpus cavernosum and the wound was subsequently closed.

**Figure 2:**
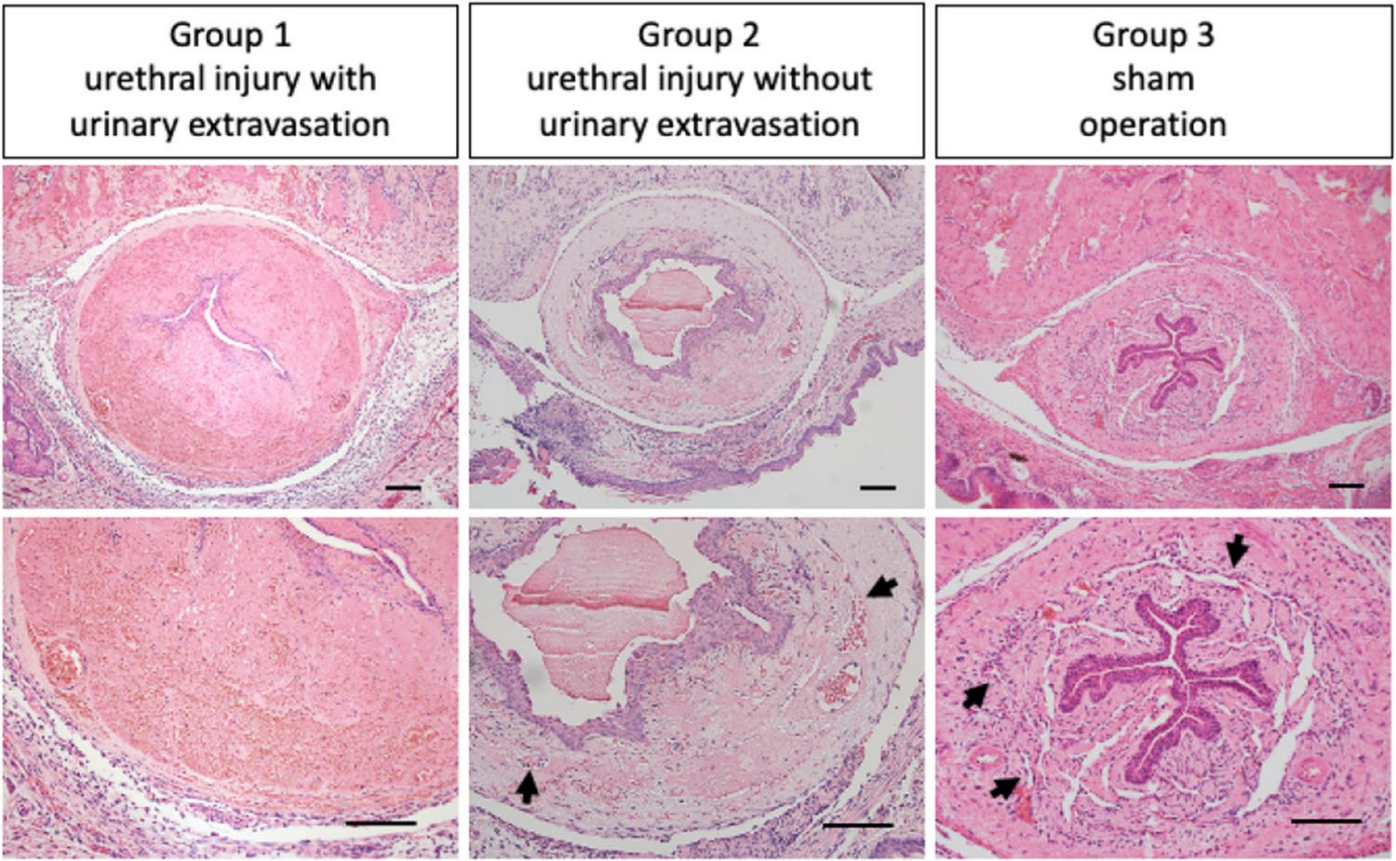
The urethral statuses for POD 3 were shown in the Hematoxylin–Eosin staining. The second row is a magnification of the first row. Severe edematous lesions of urethral mesenchyme and the narrow urethral lumen in Group1 (urethral injury with urinary extravasation) is compared with those of Group 2 (urethral injury without urinary extravasation), Group 3 (sham operation) (Scale bar: 100 µm).

**Figure 3:**
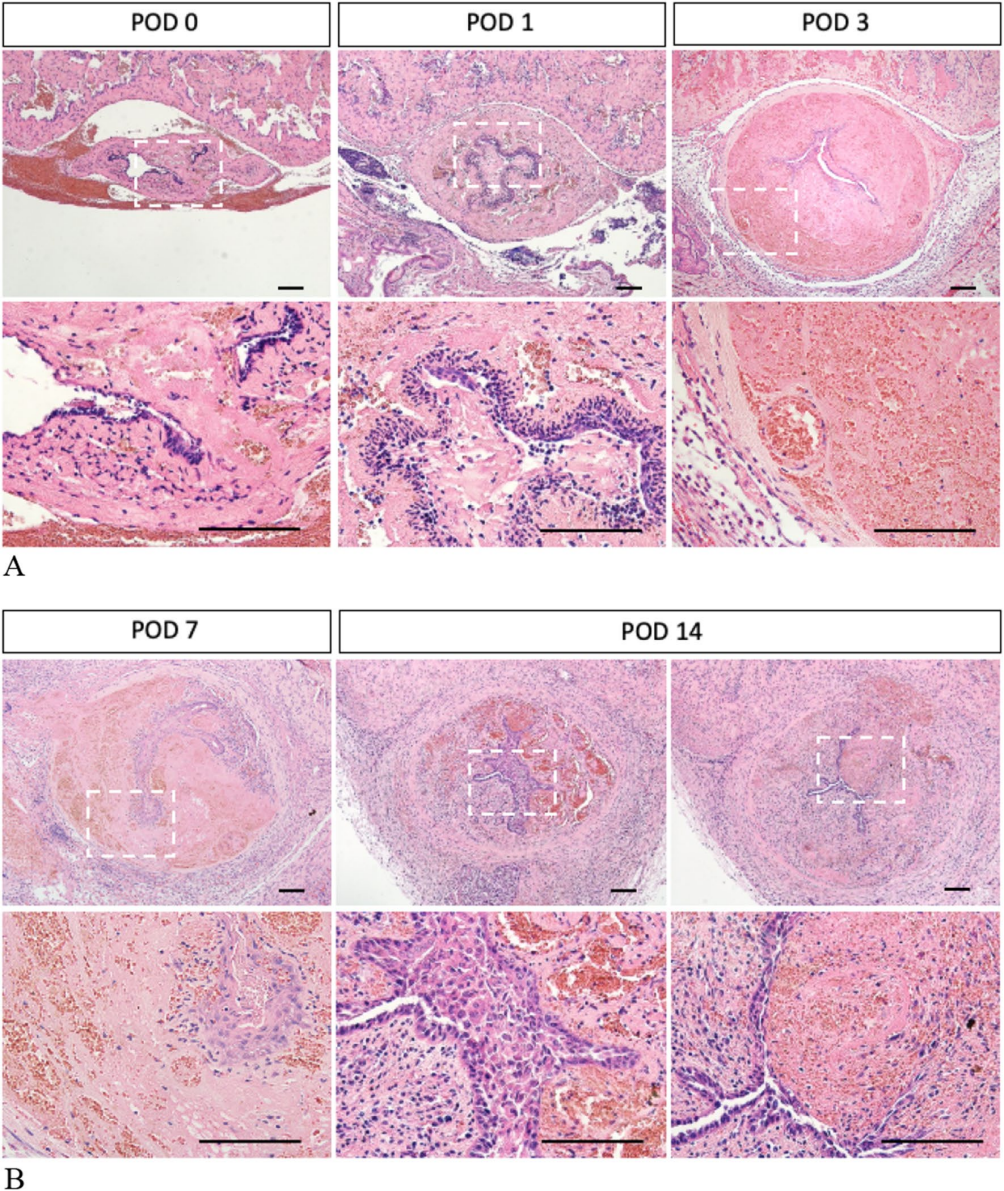
The time courses of the urethral status after urethral injury with urinary extravasation (Group1) were shown by Hematoxylin Eosin staining (Scale bar: 100 µm). The second row is a magnification of the first row. Mild inflammatory responses of urethral mesenchyme were shown at POD1 (**A**). Severe urethral mesenchymal edematous lesions continued from POD3 to POD7 (**A**, **B**). Urethral lumen has narrowed from POD 3 to POD14 (**A**, **B**). White squared regions are enlarged in the lower rows.

The time courses of the wound healing with urinary extravasation were investigated. The HE (Hematoxylin–Eosin) staining for POD (post-operative days) 3 revealed severe edematous lesions of urethral mesenchyme and the narrow lumen in Group1. However, the normal urethral mesenchyme with its lumen was maintained in Group 2, 3 (Fig. 2). The histology for Group 2 and 3 showed similar urethral mesenchymal structures. The sinusoidal structures were maintained in Group 2 and 3 (Fig. 2; black arrows). Such structures were characteristic for the corpus spongiosum penis. Acute inflammatory responses of edematous mesenchymal lesion were observed for Group 1 at POD3 (Fig. 2). Mild inflammatory responses of urethral mesenchyme without edematous lesions were shown for POD1 in Group1. Severe urethral mesenchymal edematous lesions were maintained from POD3 to POD14 in Group 1 (Fig. 3A,B; white squared regions are enlarged in the lower rows).

### The epithelial and mesenchymal histology after urethral injury and the urinary extravasation

**Figure 4:**
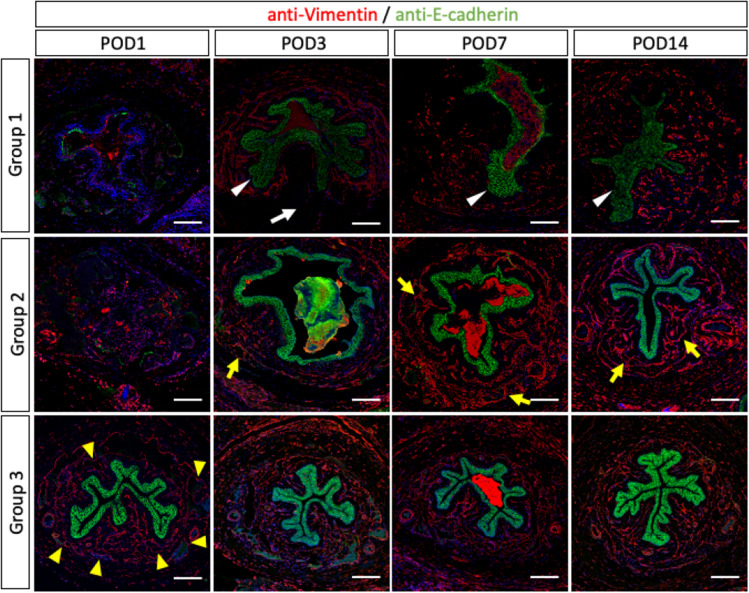
Post-surgical epithelial and mesenchymal histological structures were investigated by the expression of E-Cadherin (green) and Vimentin (red) in immune-fluorescence staining (Scale bar: 100 µm). The epithelial E-Cadherin expressions disappeared in both Group1 (urethral injury with urinary extravasation) and Group 2 (urethral injury without urinary extravasation) at POD1. As for mesenchymal histology of the Group1, the Vimentin expression disappeared at POD3 (white arrow in the second image from the left of the upper row). However, such mesenchymal expression was already recovered in POD3 in Group2 (yellow arrow in the second image from the left of the middle row). In Group1, the Vimentin expressions were gradually recovered in POD7-14, albeit the loss of mesenchymal sinusoidal structures (the third and fourth images from the left of the upper row). The urethral epithelium was thickened from POD3 (white arrowhead) in Group1.

Post-surgical epithelial and mesenchymal structures were investigated by the expression of E-Cadherin and Vimentin. In early phase of urethral injury at POD1, the histological images of Group1 were similar to those of Group2. The epithelial E-Cadherin expressions disappeared in both Group1 and 2. The Vimentin expression in the mesenchymal lesions and sinusoid structures were not maintained in both Group1 and 2 at POD1 (Fig. 4; the first image from the left of the upper and middle row). The sinusoidal structures were observed in Group 3 (Fig. 4; the first image from the left of the lower row, yellow arrowheads).

As for mesenchymal status of the Group1, the Vimentin expression disappeared at POD3 (Fig. 4; white arrow in the second image from the left of the upper row). However, such mesenchymal expression in Group2 was already recovered in POD3 (Fig. 4; yellow arrow in the second image from the left of the middle row). In case of Group2, the sinusoidal structures of the mesenchyme were maintained in POD7-14 (Fig. 4; yellow arrows in the third and fourth image from the left of the middle row). With urinary extravasation (Group1), the Vimentin expression was gradually recovered in POD7-14, albeit the loss of mesenchymal sinusoidal structures (Fig. 4; the third and fourth images from the left of the upper row).

The E-Cadherin expressions in both Groups were already recovered in POD3. However, urethral epithelium was thickened from POD3 of Group1 (Fig. 4; white arrowhead). On POD14, it was more thickened with the narrow or obstructed urethral lumen (Fig. 4; the fourth image from the left of the upper row).

### The cell proliferation for urinary extravasation was significantly increased

**Figure 5:**
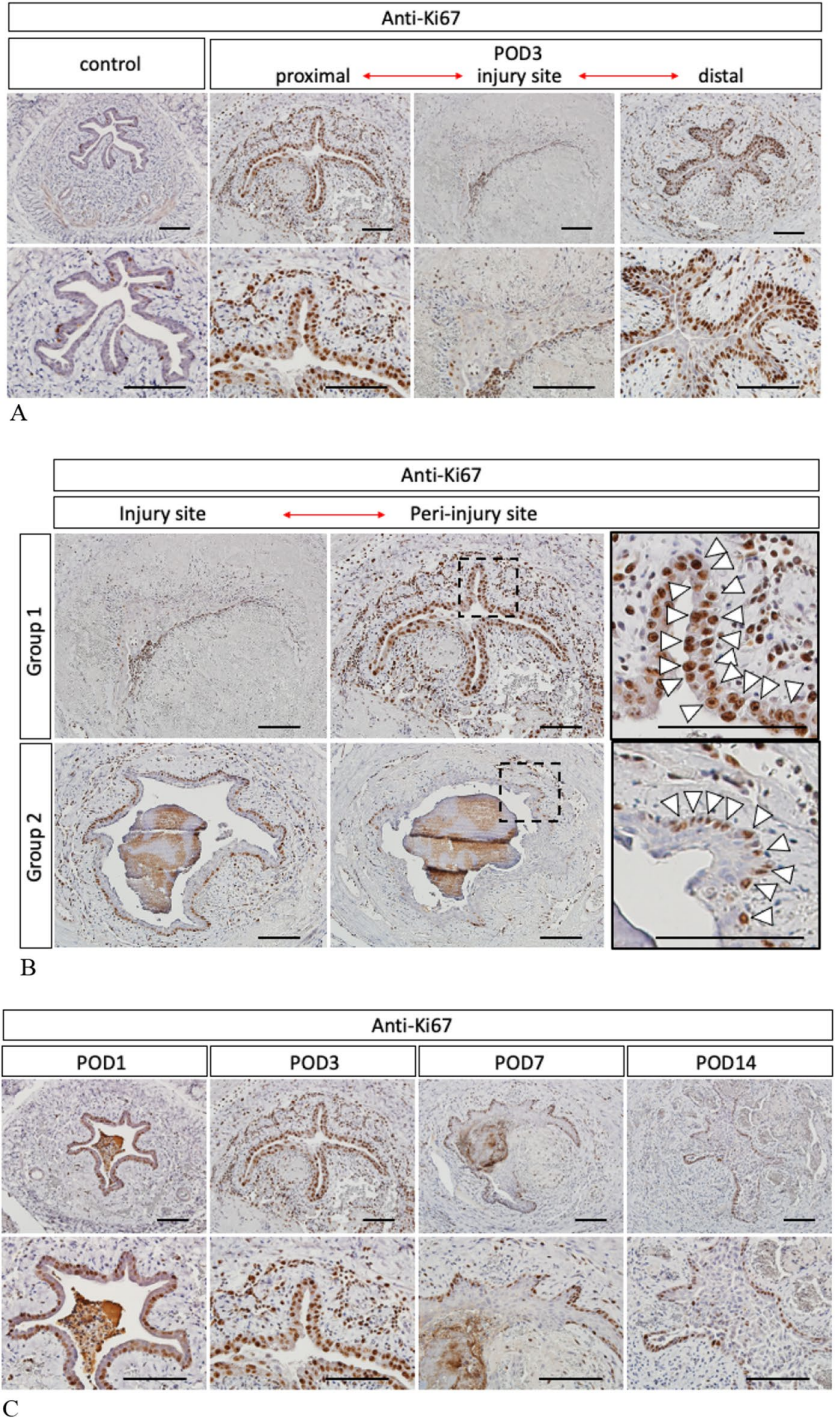
Cell proliferation in Group1 was analyzed by immunohistochemistry of anti-Ki-67 on POD3. The second row is a magnification of the first row (**A**). The significantly increased cell proliferation in Group1 was compared with that of Group2 (**B**; white arrowheads in the dot box region). The positive cells of anti-Ki-67 were positioned in the wide layers in Group 1 and those of Group 2 were positioned only in the basal side. The time course of cell proliferation in Group 1 was shown. The second row is a magnification of the first row (**C**; POD1-14) (Scale bar: 100 µm).

Cell proliferation was analyzed by immunohistochemistry of anti-Ki-67 (the prototypic cell cycle related nuclear protein). On POD3, the increased cell proliferation was detected at both of the edges of injury sites in Group1 shown by proximal or distal sites in Fig. 5A. The increased Ki-67 expressions were detected in both epithelial and mesenchymal lesions. The epithelial cell proliferation was significantly increased in the wide layers (Fig. 5A). The cell proliferation in Group1 was significantly increased compared with that of Group2 (Fig. 5B; the white arrowheads). Subsequently at POD3, the augmented cell proliferation was maintained in epithelial basal side for Group1 (Fig. 5C).

### The mesenchymal ‘spongio-fibrosis’ was induced with extravasation

**Figure 6:**
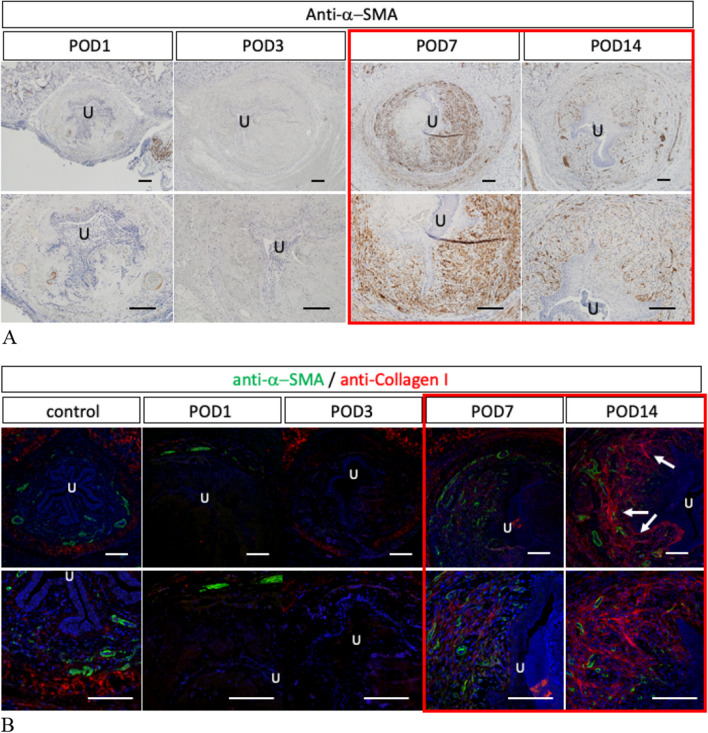
The time course of expression of α smooth muscle actin for Group1 was indicated (**A**). Its prominent expression (at POD7) and remaining expression (at POD14) were shown in red box (**A**). Post-surgical time course of expression of α smooth muscle actin (green) and Collagen I (red) was shown by immune-fluorescence staining (**B**). The prominent Collagen I expression (at POD14) and its low level of expression (at POD7) were shown in red box (**B**). “U” indicates the location of urethral lumen (Scale bar: 100 µm). The second row is a magnification of the first row.

The time course of expression of alpha smooth muscle actin (α-SMA) was investigated for Group1. The expression of α-SMA was not prominent during early phase of injury (POD1-3). Such expression was significantly increased in POD7 and decreased subsequently (Fig. 6A; red square). The expression of Collagen I was gradually increased during POD7-14 (Fig. 6B; red square). In POD14, its expression was significant and widely distributed in the mesenchymal area (Fig. 6B: white arrows).

### Live visualization of the urethral stricture with urinary extravasation

**Figure 7:**
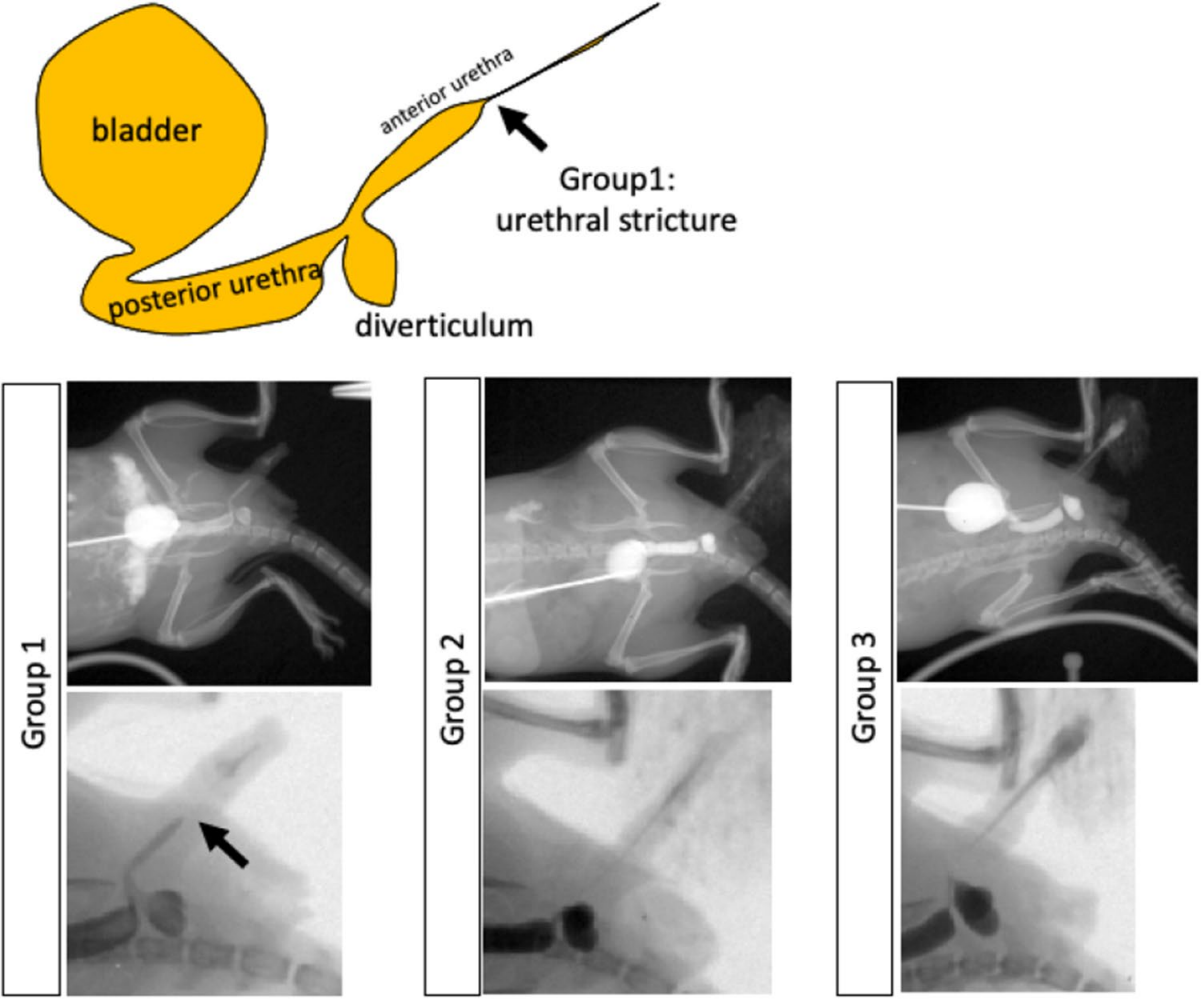
The schema of the urethral stricture in murine urethral injury model (Group 1) is shown (upper). The antegrade urethrography images in POD 3 were shown (lower). The lower row represented the magnified view of the upper row. The black arrow showed the site of urethral stricture.

To evaluate urethral status during urethral injury and urinary extravasation, we utilized not only histopathological analysis but also with urethral visualization technique which was developed previously^12^. In Group1, antegrade urethrography in POD3 showed the urethral stricture (Fig. 7; black arrow). In particular, the image of contrast agent of proximal anterior urethra was dilated and it was disappeared in the distal anterior urethra through the bladder and posterior urethra. Group2 and 3 specimens showed no urethral stricture without the urethral dilatation (Fig. 7).

## Discussion

### Elucidation of tissue responses by the exposure of extravasated fluid as an organ injury

Regarding to the post-surgical organ injury, it has been reported the exposure of the fluid such as peritonitis due to urinary or gastrointestinal perforation induces systemic or local inflammation to the surrounding tissue^13^. However, it has not been reported for the detailed tissue responses reflected by such fluid extravasation. The urethra possesses the characteristic structure termed corpus spongiosum or corpus cavernosum urethra. Corpus spongiosum is the spongy tissue containing well developed vasculatures reminiscent to the plexus structure underneath of the rectum/anus or liver sinusoids. Histological differences, vascular rich plexus or sinusoid structure exist adjacent to the ductal urethra, rectum/anus and liver parenchyma. Thus, fluid leakage to the adjacent mesenchymal tissues containing well developed vasculature is one of the general post-surgical complications. Related with such vascular rich structures, mesenchymal plexus or sinusoid has been implicated in other forms of gastrointestinal, rectal surgical treatment. Hence, it is important to investigate the wound healing process in such ductal organs^14,15^. Although the status of urinary leakage has been considered clinically important in the field of urology, its pathophysiology and mechanism have been still unknown. Regarding to the animal models, iatrogenic rabbit urethral injury models using endoscopic coagulation were reported^16–18^. However, there were no reports which describe the influence of the urine extravasation to urethra.

**Figure 8:**
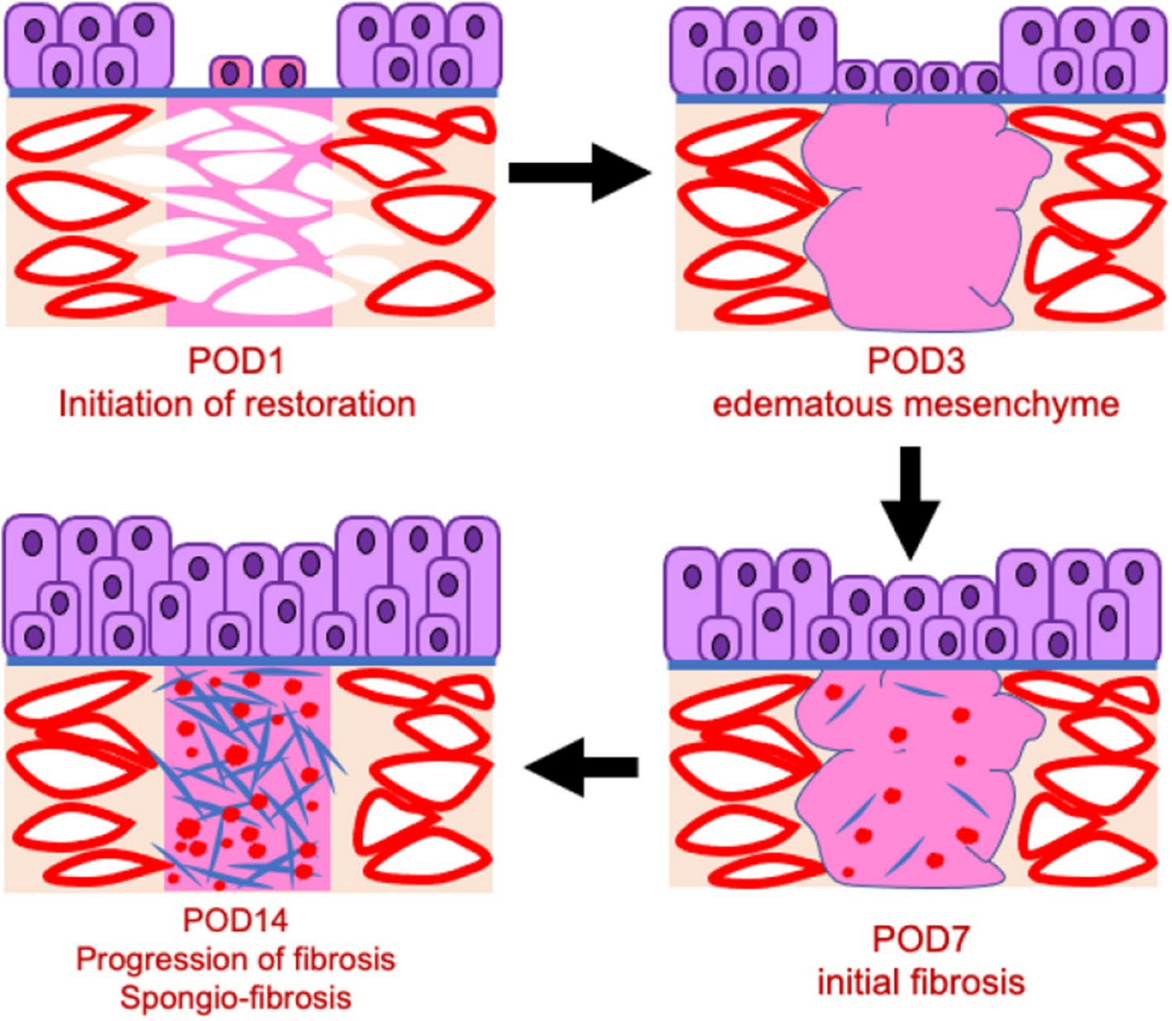
The model schema shows the status after urethral injury with urinary extravasation. The severe edematous lesions of urethral mesenchyme in post-operative days 3 are represented as the pink spread lesion (the upper right image). Red sinusoidal or dot lesions are represented by the positive lesion of anti-Vimentin staining. The blue sharp rod lesions were represented as the positive fibrous Collagen I expression (the images of lower row). The status and the time course of spongio-fibrosis are indicated (the images toward the left low one).

Urethral injury with urinary extravasation influences urethral epithelium, leading to the urethral thickening and stricture (Fig. 8; the thickened epithelia in the lower row). A previous review reported that urethral stricture was shown by the injury of urethral epithelium and urine exposure^9^. This study showed the both Groups of the urethral injury with or without the urine exposure have suffered the urethral epithelial damages with reduced E-Cadherin expression. In the Group1 of the urethral injury with urine exposure, urethral epithelium was regenerated with thickening and narrowed urethral lumen, whereas urethral injury without urine exposure regenerated significantly. The critical effects were only observed in the cases of extravasating urine into the urethral mesenchyme and epithelia. Post-surgically, accelerated cell proliferation was reported in case of rat urethral injury models^19^. The cell proliferation was accelerated in the edge of the injury sites, similar to other urinary tract wound healing^20,21^.

In urethral epithelium, the cell proliferation took place in the basal side. However, the epithelial cell proliferation was significantly increased widely in the cases with urinary extravasation (Fig. 5B). The exposure to the urine, triggered the subsequent excessive epithelialization^22^. The augmented cell proliferation in all epithelial layers could be a characteristic landmark for subsequent phenotypes.

### The structure and expression characters of sub-epithelial layer or lamina propria of mouse urethra

The urethral epithelium and corpus spongiosum were well known structure histopathologically^23–25^. As for detailed anatomy of human urethra, there were only a few reports^26–30^. In the previous reports of human corpus spongiosum, there were few vascular structures reported between the urethral epithelium and the corpus spongiosum. However, the detailed anatomy of murine urethra has not been understood yet^31^. In the current study, the clear zone between the expression area of E-Cadherin and Vimentin was detected. Previous reports termed this area as sub-epithelial layer or lamina propria (LP). However, the borders of sub-epithelial layer have not been revealed yet^1,23^. For the first time, our results suggested the collapse of sub-epithelial layer (or LP) resulted in the inflammatory reaction of epithelium and mesenchyme (Group1, Fig. 1, 3A). LP is known to play the important role in the intestinal immune system^32^. Significant number of VEGFR1 (vascular endothelial growth factor receptor 1) positive cells in the layer were detected at POD1 (data not shown). The epithelium already regenerated on POD3. We suggested the reaction of the injury was detected in the layer at early phase of injury.

In the bladder, the LP lies between the basement membrane of the mucosa and the detrusor muscle and is composed of an extracellular matrix containing fibroblasts, adipocytes, interstitial cells^33^. In addition, the disease such as interstitial cystitis is triggered by the mesenchymal inflammation. The roles of the LP in bladder function have still not been definitively established, though it has been suggested for enabling adaptive changes to increasing volumes^33^. The role of sub-epithelial layer and corpus spongiosum should be further compared from the viewpoints of subsequent tissue reactions from layers of epithelia to the underlying mesenchyme.

### The first murine model for urinary extravasation

The severe inflammation and the mesenchymal edema in early phase of urethral compression injury were induced by urinary extravasation. There have been no reports of wound healing process in urethral injury and urinary extravasation. Hence, this study first reported the essential roles of urinary exposure in urethral injury. Furthermore, the study revealed the time course of urethral healing process. This process eventually led to spongio-fibrosis which had not been reproduced yet as a model^8,34^.

The bladder does not possess a spongy structure unlike the urethra. The bladder prevents leakage by allowing extension and contraction and distributing pressure across its broad wall. Mechanical perforation of the urinary bladder of Sprague–Dawley rats and subsequent administration of phenacetin induced urothelial hyperplasia. However, no pathological changes were found in the rat bladders only submitted to mechanical perforation or phenacetin treatment^35^.

In contrast, the urethra prevents leakage by the tight sealing of the epithelium and the supporting structure of the corpus spongiosum, which can tolerate the high urinary pressure. The responses of urethra and bladder by the urinary extravasation thus show different symptomatic characteristics. Further researches are necessary to investigate such differences.

Since various mutant model mice are available and experimental visualization has also been established, further combinatory studies are expected by mouse studies. Mouse models will be generally more created and tested and genetically modified mice model will be also increasingly utilized. The current results will lead to the exploration of the pathogenesis of complications after pediatric urologic surgery and development of treatment strategy.

## Methods

### Mice

Mice were purchased from CLEA Japan, Inc. (Tokyo, Japan). The adult ICR male mice (8–12 weeks) were utilized and bred under controlled temperature (21 °C) with a 12:12 h light–dark cycle. The protocol was approved by the Animal Ethics Committee of Wakayama Medical University (Approved Number 1061). All methods were performed in accordance with the relevant guidelines and regulations. The study was carried out in compliance with the ARRIVE guidelines (Fig. S1).

### Murine urethral injury (trauma) and urinary extravasation model

The general anesthesia was performed by Medetomidine, Midazolam, Butorphanol and was administered trans-peritoneally. The samples were collected on post-operative days (POD) 1, 3, 7, 14. Each experiment was performed by the three groups. Mice were sacrificed after collecting tissue samples under the general anesthesia. The mice which survived to the schedule date were utilized for the subsequent study (survival rate 88.5%; n = 52). About sample size, in Group1 (urethral injury with extravasation), the number of mice at POD1 were 5, POD3 (n = 5), POD7 (n = 4), POD14 (n = 4). In Group2 (urethral injury without extravasation), those were at POD1 (n = 2), POD3 (n = 4), POD7 (n = 4), POD14 (n = 3); In Group3 (sham operation), POD1 (n = 2), POD3 (n = 4), POD7 (n = 3), POD14 (n = 3). Samples with POD0 were collected for Group1 only for an immediate postoperative evaluation (n = 2). A total of 52 mice were utilized. To minimize potential differences that arise between treatment groups during the course of the experiments, all surgical operation was done by the same person as standardization. The animal care staffs were unaware of allocation groups to ensure that all animals in the experiment are handled, monitored and treated in the same way. There was no therapeutic intervention of any kind after surgical operation and qualitative evaluation was thus conducted utilizing histological staining or X-ray imaging. The same method was utilized for all the histological staining.

### Histopathological, immunofluorescence and immunohistochemical staining

The operated mouse tissues were fixed overnight in 4% (wt/vol) paraformaldehyde/phosphate buffered saline. Samples were subsequently dehydrated and embedded in paraffin. The 6 µm thick sections were prepared. Hematoxylin Eosin (H.E.) staining was performed as the previously described^12^. Imaging was visualized using the microscope (OLYMPUS BX51, DP80). For immunofluorescence, the antigen retrieval was performed with citrate buffer, autoclaved at 121 °C, 1 min. The primary antibodies were utilized as follow; anti-E-Cadherin (mouse 1/100, 610182 BD Transduction Laboratory), anti-Vimentin (rabbit 1/100, ab92547 Abcam), anti-α-SMA (mouse 1/200, A2547 Sigma-Aldrich), anti-Collagen I (rabbit 1/100, ab270993 Abcam). The secondary antibodies were Goat anti-Rabbit IgG (H + L) Alexa Fluor 546 (A-11010), Goat anti-Mouse IgG (H + L) Alexa Fluor 488 (A-11001) Cross-Adsorbed Secondary Antibody. Hoechst 33342 (1/1000, B2261 Sigma-Aldrich) was utilized for counterstaining. Visualization was performed using the confocal laser scanning microscope (Zeiss LSM 700). For other immunohistochemical staining, the antigen retrieval was performed with citrate buffer, autoclaved at 121 °C, 1 min. The primary antibodies were utilized as follow; anti-Ki67(rabbit 1/100, NCL-Ki67p Leica), anti-α-SMA (mouse 1/200, A2547 Sigma). The Goat Anti-Mouse (1/200, BA-9200 Vector Laboratories), Anti-Rabbit (1/100, BA-1000 Vector Laboratories) IgG Biotinylated Antibody were utilized as the secondary antibodies. The signal amplification using Vectastain ABC kit (Vector Laboratories) was performed. The counterstaining was performed using Hematoxylin staining.

### Murine antegrade urethrography by in vivo imaging system

IVIS LUMINA XR (Caliper Life Sciences Inc. Hopkinton, MA) was utilized as imaging device. The settings were described as our previous report^12^. In brief, the mouse was placed oblique position and antegrade urethrography was performed using contrast agent (Iopamiron 375; Iopanidol, Bayel AG, Osaka, Japan).

## Supplementary Information

Supplementary Information 1.

## Data Availability

The datasets used and/or analyzed during the current study available from the corresponding author on reasonable request.
