# Supplementary figures and images for: Wound healing responses of urinary extravasation after urethral injury
Source: Sci Rep. 2023 Jun 30;13:10628. doi: 10.1038/s41598-023-37610-2 (PMC10313654; doi:10.1038/s41598-023-37610-2)

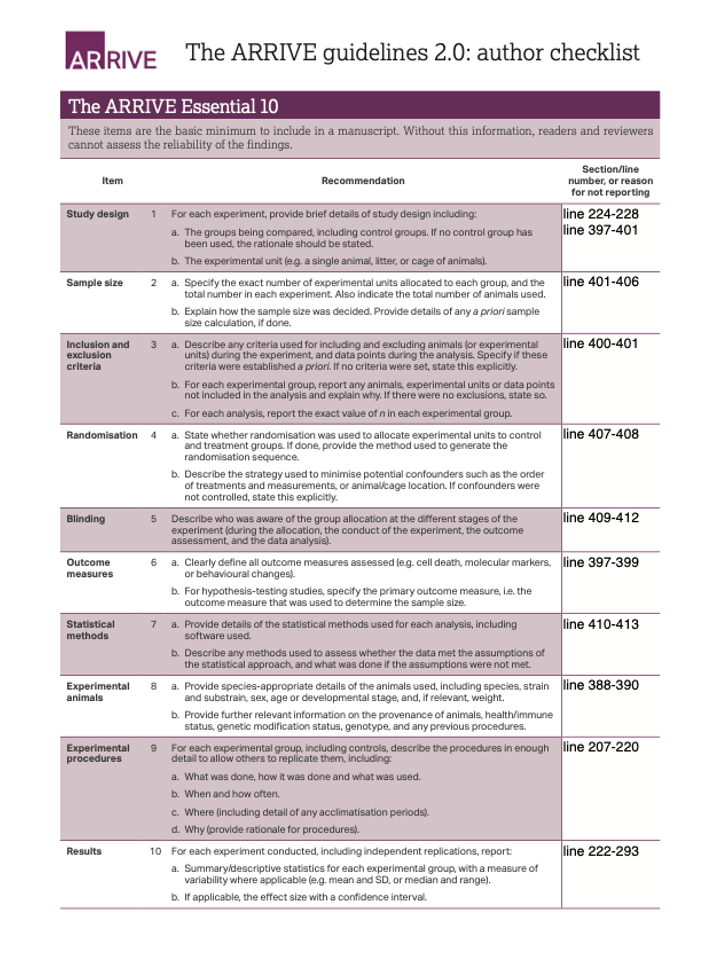

Supplement: Supplementary file 1 — Supplementary Information 1. [file 41598_2023_37610_MOESM1_ESM.tiff]
